# Spermatogenesis and Cryptorchidism

**DOI:** 10.3389/fendo.2014.00063

**Published:** 2014-05-01

**Authors:** Giovanni Cobellis, Carmine Noviello, Fabiano Nino, Mercedes Romano, Francesca Mariscoli, Ascanio Martino, Pio Parmeggiani, Alfonso Papparella

**Affiliations:** ^1^Paediatric Surgery, Salesi Children’s Hospital, Università Politecnica delle Marche, Ancona, Italy; ^2^Paediatric Surgery, Department of Paediatrics, Faculty of Medicine, Second University of Naples, Naples, Italy

**Keywords:** cryptorchidism, undescended testes, spermatogenesis, germ cells, testicular cancer, orchidopexy

## Abstract

Cryptorchidism represents the most common endocrine disease in boys, with infertility more frequently observed in bilateral forms. It is also known that undescended testes, if untreated, lead to an increased risk of testicular tumors, usually seminomas, arising from mutant germ cells. In normal testes, germ cell development is an active process starting in the first months of life when the neonatal gonocytes transform into adult dark (AD) spermatogonia. These cells are now thought to be the stem cells useful to support spermatogenesis. Several researches suggest that AD spermatogonia form between 3 and 9 months of age. Not all the neonatal gonocytes transform into AD spermatogonia; indeed, the residual gonocytes undergo involution by apoptosis. In the undescended testes, these transformations are inhibited leading to a deficient pool of stem cells for post pubertal spermatogenesis. Early surgical intervention in infancy may allow the normal development of stem cells for spermatogenesis. Moreover, it is very interesting to note that intra-tubular carcinoma *in situ* in the second and third decades have enzymatic markers similar to neonatal gonocytes suggesting that these cells fail transformation into AD spermatogonia and likely generate testicular cancer (TC) in cryptorchid men. Orchidopexy between 6 and 12 months of age is recommended to maximize the future fertility potential and decrease the TC risk in adulthood.

## Introduction

Undescended testis or cryptorchidism is the most common genital abnormality in boys. The prevalence of cryptorchidism in full-term newborns range between 1 and 3%, reaching 30% in prematures (1–3). The pathology is bilateral in about 20% of the cases. About 80% of undescended testes are palpable and 20% are non-palpable (3–5). Palpable undescended testes are located along the inguino-scrotal region. Non-palpable testes may fall into one of the following categories: intra-abdominal location, agenesis, intrauterine demise, or inguinal location caused by dysplasia or atrophy. It is important to differentiate the true cryptorchidism from the retractile testis, which is a normal finding and usually it does not require surgical treatment. Acquired cryptorchidism has been observed when the retractile testis ascent in the inguinal canal during the infancy (ascending testis).

The main risk factors for the cryptorchid testis are infertility and testicular cancer (TC).

The risk of infertility in adulthood is more significant in patients with bilateral undescended testes (6). Approximately 10% of the infertile men have a history of cryptorchidism and orchidopexy (7). Azoospermia is evident in 13% of unilateral cryptorchidism and increase to 89% in untreated bilateral cryptorchid patients (8), although boys with one undescended testis have a lower fertility rate, they have the same paternity rate as boys with bilateral descended testes. Boys with bilateral undescended testes have a lower fertility and a paternity rate (9). In some studies, patients with unilateral cryptorchidism had normal spermatogenesis, suggesting that additive detrimental factors may be responsible for impaired fertility. The studied mechanisms of the infertility in cryptorchidism are multiple (7). The hyperthermia, between 35 and 37°C rather than 33°C, evoked by the abnormal position of the testis may respond for the impaired spermatogenesis. Anatomical congenital anomalies associated to undescended testis as testis–epididymis disjunction or iatrogenic lesions of vas and testis during orchidopexy may also contribute to infertility. Retrospective studies in infertile patients with history of cryptorchidism have demonstrated an increased incidence of anti-sperm antibodies which is more evident in pubertal age (1, 8). Sinisi et al. showed that cryptorchidism may elicit an autoimmune response against sperm antigens in childhood independent of testis location and orchidopexy (1).

It is known that undescended testes, if untreated, lead to an increased risk of TC, usually seminomas (10), arising from mutant germ cells. TC is a solid neoplasm that has an incidence of 1% of all cancers in men and is the most common between 20 and 30 years of life (11, 12). Boys with an undescended testis have a 20-fold higher risk to develop a TC and about 10% of the cases of TC develop in men with a history of cryptorchidism (13).

In this review, we focus on the current knowledge about the abnormal germ cell development in the undescended testes and its possible relationship with the impaired spermatogenesis and TC in adulthood. In the second section of this review, we discuss the treatment of cryptorchidism and the possible role of the early orchidopexy in the prevention of both infertility and cancer.

## Germ Cell Development, Infertility, and Testicular Cancer in Cryptorchidism

The germ cell development and its modification in cryptorchidism have been recently matter of many researches (2, 14, 15).

Spermatogenesis is the process by which sperm cells are produced. In men, it starts at puberty, resulting from the increased levels of gonadotropins and testosterone. It is a complex process including sequential steps of mitosis, meiosis, and differentiation. In each of these steps, endocrine, paracrine, and autocrine factors are involved (16). Spermatogenesis takes place in the seminiferous tubule: here germ cells are organized from the base of the tubule to the lumen and progressively develop from spermatogonia to spermatids. In the last step, spermatids differentiate through morphological transformation into spermatozoa (spermiogenesis) (17) which are finally released from the Sertoli cells into the lumen of the seminiferous tubule (spermiation).

However, germ cell development is an active process. It starts during the first years after birth when neonatal gonocytes change into adult dark (AD) spermatogonia. These are stem cells and have a dark nucleus that specifically characterize them from the other germ cells. Therefore, AD spermatogonia do not directly take part to sperm production; nevertheless, they ensure a supply of stem cells for spermatogenesis. Indeed, AD spermatogonia replicate to produce adult pale (AP) spermatogonia, with light nuclei. These cells produce by mitosis the type B spermatogonia which further divide and differentiate into primary spermatocytes which are already evident in the testes of children 4 years of age (2, 18). Two sequential meiotic divisions and spermiogenesis lead to final development of round spermatids and spermatozoa, respectively (19).

Several data suggest that AD spermatogonia form between 3 and 9 months of age. This developmental cycle needs normal testicular hormones and the optimal scrotal temperature of 33°C (20, 21). The hormonal regulation of these changes is not fully understood, with evidence for a possible role of gonadotropins and androgens. Not all the neonatal gonocytes transform into AD spermatogonia. The remaining gonocytes undergo involution by apoptosis. Genetic aberrations and environmental conditions influence these processes.

The failure of transformation of gonocytes into AD spermatogonia may produce infertility in boys.

Hadziselimovic and Herzog (15) have demonstrated that the process of transformation of neonatal gonocytes into AD spermatogonia during the first year of life is crucial for male fertility. The inhibition of this process in undescended testis leads to a deficient pool of stem cells for post pubertal spermatogenesis and infertility. Moreover, in undescended testes, germ cells loss starts at 6 months of age. Testicular biopsies at time of orchidopexy confirmed the importance of AD spermatogonia for fertility in cryptorchid patients. Tasian and coworkers (22) observed greater germ cell depletion in abdominal testes compared with palpable testes and a progressive germ cell loss for each month the testes remain undescended.

It is very interesting to note that the intra-tubular carcinoma *in situ* (CIS) in the second and third decade has enzyme markers similar to neonatal gonocytes as placental alkaline phosphatase expression, suggesting that these cells, that fail to develop in AD spermatogonia at 3–9 months of age, are the origin of cancer in cryptorchid men (23). Studies have suggested that the precursor cells of testis cancer, testicular CIS, are similar to fetal gonocytes. A current hypothesis (2) is that, due to the high temperature anomaly of undescended testis, an abnormal apoptosis allows some gonocytes to persist and become CIS with progressive mutation and/or cellular unbalance, and eventually malignancy in adulthood. These abnormal gonocytes are kept in a defined environment “suspended animation” in the germ-line and, due to the accumulation of mutations, may undergo transformation becoming the source of the CIS (2, 21, 24).

The etiology accepted for germ cell carcinoma remains unknown, although disturbances in the microenvironment provided by the Sertoli and Leydig cells may play an important role. In fact, spermatogenesis is strictly controlled and depends on a succession of signals supplied from the local environment (11, 25, 26) and Leydig cells, next to their steroidogenic function, during development express the insulin-like-3 gene (INSL3), which is responsible for gubernaculum maturation and testicular descent (27). A specific association of mutations in INSL3 with cryptorchidism has been described but its possible role in TC development and infertility needs to be clarified (28).

Olesen et al. linked the development of TC not only with cryptorchidism but also with other urogenital anomalies such as hypospadia (29). In fact, epidemiological studies in males who presented fertility problems tend to lean toward an enhanced risk of testicular germ cell tumor (30). The development of TC is associated with many chromosomal abnormalities and this raises the problem for close monitoring of these patients. Kanetsky et al. (31) demonstrated common genetic variants associated to an increased risk of testicular germ cell cancer (TGCC) and found that seven markers at 12p22 within KITLG (c-KIT ligand) reached genome-wide significance. This gene has been involved in several aspects of primordial germ cell development, migration, and survival (32).

Concerning the development of the urogenital sinus and particularly the testis, the impacts of endocrine disruptors have been fairly well described on human and experimental models (33–35). This is especially true for hypospadia, cryptorchidism, and infertility; but the link with TGCC has to be explained. The unbalanced equilibrium between the estrogen and androgen levels *in utero* is hypothesized to influence the risk of TC. Thus, mutations in testosterone gene expression may change the level of testosterone *in vivo* and hypothetically the risk of developing TC (36).

As discussed before, hormonal regulation is very significant in the development of the germ-line. Beside the importance of fetal development, it seems that puberty should be an important moment, when hormone levels reach optimal concentrations for the secondary sex characters development. It has been shown that sperm agglutinating antibodies appear in young boys with cryptorchidism and they are more prevalent during puberty (1). This also coincides with the appearance of TGCC, as men affected are between 15 and 35 years old, suggesting that puberty and probably the increase in hormone concentrations should be central issues (37).

## Hormonal and Surgical Treatment of Cryptorchidism

The goals of treatment of cryptorchidism are mainly two: preserve fertility and reduce the risk of neoplastic disease. Last but not the least, treatment allows the testicular self-examination for an early diagnosis and detection of TC.

Hormonal treatment with human chorionic gonadotropin (hCG) or gonadotropin-releasing hormone (GnRH) may be initially administered for cryptorchidism because it should promote the testicular descent (38). The theoretical basis for its use is to stimulate the Leydig cells to produce testosterone, inducing inguinal–scrotal testicular descent. Potential harmful effects of hormonal treatment on the developing testes, including apoptosis, inflammation, and reduced number of germ cells are still under study. In addition, there are reports which suggest that the hormonal stimulation in infancy may be damaging to the testes (39). It has been observed, in hCG-treated rats, a poor differentiation of the seminiferous epithelium, with high Leydig cell evidence and increased inter-tubular eosinophilic material (40). These experimental data emphasize the possible negative outcome of hormone therapy on germ cell line and its main action on Leydig cells. The increased synthesis of vascular endothelial growth factor (VEGF), determined by hCG therapy also highlights the increased cell permeability causing interstitial edema. The role of VEGF on spermatogenesis is unclear. Several findings have revealed several inhibitory effects of VEGF on spermatogenesis (40, 41).

Considering the poor efficacy and the possible adverse effects of hormonal therapy, surgery must be preferred (42).

Orchidopexy is the cornerstone of cryptorchidism treatment. Inguinal operation is the standard approach for palpable testis. Laparoscopy is the gold standard technique for both diagnosis and treatment of non-palpable testes (3–5). Early surgical treatment may preserve fertility. Orchidopexy is commonly performed before 2 years of age and increasing research suggest that surgery before 1 year of age may permit the normal spermatogenesis by preventing degenerative changes of the testes and germ cell loss (22, 43). However, early orchidopexy does not guarantee normal fertility in adulthood. Hadziselimovic showed that despite orchidopexy before 6 months of age, up to 35% of boys will grow up to be infertile regardless of the normal total germ cell count on testicular biopsies performed at the time of orchidopexy (44). The current practice for the acquired cryptorchidism is to operate at diagnosis, by a scrotal approach, although the prognosis seems to be better than congenital cryptorchidism considering the normal development and apoptosis of the germinal cells during the first year of life.

Since the link between cryptorchidism and TC seems to be an abnormal development of the primary germ-line, any attempt to normalize this process, as early surgery, will permit a normal growth of germ cells, thereby avoiding cancer as well as oligospermia or azoospermia. However, it should be mentioned that some studies failed to demonstrate a correlation between the time of surgery and cancer risk (45). A systematic review and meta-analysis of the literature by an American group has concluded that prepubertal orchiopexy may decrease the risk of malignancy and that early surgical intervention is indicated in children with cryptorchidism leading to a better growth of the testis (46).

## Conclusion

Cryptorchidism is a risk factor for infertility and TC in adulthood. To date, orchidopexy is recommended between 6 and 12 months of age. The aim of an early surgical intervention is to prevent the abnormal germ cell development and ultimately decrease the risk of infertility and malignancy in adulthood.

## Conflict of Interest Statement

The authors declare that the research was conducted in the absence of any commercial or financial relationships that could be construed as a potential conflict of interest.
